# Predicting cardiovascular disease risk from gut microbial genes

**DOI:** 10.1128/mbio.01970-23

**Published:** 2023-10-16

**Authors:** Jan Claesen, J. Mark Brown

**Affiliations:** 1 Department of Cardiovascular and Metabolic Sciences, Lerner Research Institute of the Cleveland Clinic, Cleveland, Ohio, USA; 2 Center for Microbiome and Human Health, Lerner Research Institute of the Cleveland Clinic, Cleveland, Ohio, USA; 3 Department of Molecular Medicine, Cleveland Clinic Lerner College of Medicine of Case Western Reserve University, Cleveland, Ohio, USA; 4 Department of Cancer Biology, Lerner Research Institute of the Cleveland Clinic, Cleveland, Ohio, USA; University of Kentucky, Lexington, Kentucky, USA

**Keywords:** microbiome, metabolism, cardiovascular disease

## Abstract

Gut bacteria-driven production of trimethylamine (TMA) is strongly associated with cardiovascular disease. Borton et al. (mBio 14:e01511-23, 2023, https://doi.org/10.1128/mbio.01511-23) introduce the Methylated Amine Gene Inventory of Catabolism database (MAGICdb), comprehensively cataloging pathways involved in TMA metabolism. By integrating transcriptomics, proteomics, and metagenomic data, this work identifies key bacterial players in the process and can link gut microbial gene content to fecal TMA concentrations. This work shows that methylated amine metabolism is a keystone microbiome process carried out by a small proportion of the community. Proatherogenic pathways are more widely distributed among the gut microbiota, and new TMA-reducing genera were identified that might offer new potential for probiotic strategies or targeted microbiome interventions. Remarkably, MAGICdb’s power to predict cardiovascular disease risk matches an approach using more traditional lipid risk factors. This open source will be a valuable tool for the community to link methylated amine metabolism to gut microbiome-related human health conditions.

## COMMENTARY

The strong association between gut microbial production of trimethylamine (TMA) and the host associated co-metabolite trimethylamine N-oxide (TMAO) and atherosclerotic cardiovascular disease (ASCVD) is well established (1, 2). In fact, the gut microbial TMAO pathway is now considered one of the best examples of how gut microbe-derived metabolites can have a profound impact on host physiology and disease (1, 2). TMA is formed by select members of the gut microbiome using dietary quaternary amines such as choline, carnitine, glycine betaine, and butyrobetaine as substrates (3). Three main types of bacterial enzymes have thus far been identified that catalyze these catabolic reactions. The choline TMA lyase CutC is a glycyl radical enzyme that acts in concert with its activator CutD to cleave the C-N bond in choline, producing TMA and acetaldehyde. The genes for this pathway occur broadly yet discontinuously across Firmicute, Actinobacterial, and Proteobacterial genomes. Carnitine and butyrobetaine are catabolized to TMA + malate, and TMA + succinate, respectively, in an aerobic reaction by Rieske-type oxygenase/reductase enzymes of the CntA/B and YeaW/X families, which are typically found in Gamma- and Betaproteobacteria as well as a few Firmicutes. Glycine betaine is reduced by homologs of GrdI resulting in the production of TMA and acetate. TMA is taken up into systemic circulation, and upon reaching the liver, it is oxidized to the proatherogenic compound trimethylamine N-oxide by flavin-containing monooxygenase 3. TMAO contributes to an increased atherosclerotic plaque formation by altering cholesterol and lipid metabolism, as well as inducing vascular inflammation. Drugging the TMA pathway has thus far mainly focused on the bacterial enzyme choline TMA-lyase CutC. Several small-molecule substrate analogs with inhibitory activity have been developed, including 3,3-dimethyl-1-butanol, a cyclic choline analog, and halomethylcholines (4–6).

While it is important to characterize and pharmacologically target the catabolic process resulting in gut bacterial TMA production, the paper by Borton et al. highlights the importance of taking into account beneficial bacterial metabolism (7). Indeed, recently trimethylamine methyltransferase (MTTB) family enzymes from gut methanogens have been characterized that are involved in reducing TMA levels by either indirectly demethylating the quaternary amine substrates or directly catalyzing subsequent demethylation reactions of TMA itself, resulting in production of ammonia (8). The extent to which these “non-TMA” pathways decrease the bioavailable levels of TMA and whether this effect is substantial enough to impact host physiology and ASCVD risk was underappreciated.

Borton et al. addressed these questions by generating the Methylated Amine Gene Inventory of Catabolism database, which conveniently abbreviates to MAGICdb (7). This database encompasses the four main pathways for TMA production described to date, starting from choline, glycine betaine, carnitine, and butyrobetaine. To account for degradation of these substrates or TMA itself, genes encoding MTTB enzymes were included in MAGICdb, as these could cause a shunt from the trimethylated amine substrate pool.

Next, the authors proceeded to look for a connection between fecal TMA concentrations and their methylated amine (MA) metabolism gene content in a cohort of healthy individuals. They performed metagenomics sequencing at much greater depth compared to more traditional approaches and uncovered twice as many MA metabolism genes. It was notable that MA metabolism is variably distributed across individuals, but it seems not to be correlated with sex, BMI, or lifestyle. Among the specific genes included in MAGICdb, the proatherogenic cntA/yeaW and non-TMA genes, myyB and mtxB, were found most associated with higher and lower fecal TMA concentrations, respectively. Taken individually, none of the genes were sufficient to predict fecal TMA concentrations. However, when the relative abundances of all pathways were taken into account they did correlate well, underscoring the importance of the cumulative analysis encompassed in MAGICdb. One potential caveat that may have contributed to the low correlation between metagenomic and metabolomic data sets is the fact that TMA rapidly dissipates during sample collection and storage due to its highly volatile nature. Even with this well-known challenge in studying volatile amines in stored biological samples, the metabolic predictions provided by MAGICdb will be highly invaluable for future experiments defining the key bacteria that shape circulating TMA and TMAO levels *in vivo*.

From the sequencing data in the healthy cohort, 1,436 unique metagenome-assembled genomes were constructed, and 21 of these contained either proatherogenic or non-TMA-associated MA genes. One genome from an Anaerovoracaceae member contained both TMA-producing and TMA-depleting genes. Even in the healthy cohort, proatherogenic bacteria, including *Enterocloster* and *Dorea*, were found in far greater abundance, as well as more widely distributed across individuals compared to the non-TMA-producing bacteria such as *Bilophila*. Overall, bacteria that encode MA metabolic genes made up under 3% of gut microbial genomes, highlighting their relative rareness. The authors conclude that gut microbial MA metabolism is keystone process, it is important for host physiology despite being encoded in only low abundance members of the gut microbiota.

This study also provides a perfect example of the limitations of relying solely on taxonomy-based predictions based on 16S rRNA sequencing. While most bacterial genera contain members that are either proatherogenic or non-TMA producing, *Bilophila* is a notable exception. The large majority of *Bilophila* (about 97%) is non-TMA producing, ~1.5% is solely proatherogenic, and less than 1% could both produce as well as metabolize TMA. Furthermore, only half of *Escherichia* genomes and 19% of *Blautia* genomes contained proatherogenic MA metabolism genes. Hence, it is important to consider actual microbiome function using metagenomics, as there is a clear delineation between strains within a certain genus that do or do not have the genes required for MA metabolism.

The presence and abundance of MA metabolism genes in a metagenomics data set do not necessarily reveal which ones are active and matter for biological function. The authors addressed this obstacle by intersecting sequencing MAGICdb with transcriptomics and proteomics data from bacterial communities grown in fecal reactors, as well as published human cohort studies. In the anoxic, polymicrobial reactor cultures, choline and glycine betaine were identified as the main proatherogenic precursors. Proatherogenic metabolism was found more generally widespread across different microbial genera, whereas demethylation activity was performed primarily by *Lactonifactor* and *Eubacterium*. Addition of different MA substrates to the fecal reactor identified one single *Eubacterium* MyxB enzyme, which likely was responsible for demethylation of all tested substrates, indicating that strains of this genus could be good candidates for prioritization as TMA-lowering probiotics. Analysis of the multi-omics human cohort data put Clostridial genera, such as *Dorea,* on the map as prominent TMA producers alongside better-known culprits among the Gammaproteobacteria.

The value of MAGICdb extends beyond cataloging, as the database performed impressively at predicting cardiovascular disease with high accuracy. A previously published ASCVD study was reanalyzed with MAGICdb, which features increased sampling capabilities and includes additional MA genes over the original analysis. This inclusion of the full MA gene repertoire, as well as taking into account the diversity of MA genes across the patient cohort significantly increased the predictability. In particular, ASCVD individuals were found to have an increased relative abundance of proatherogenic genes, concomitant with a depletion of the *mttB* non-TMA gene. Leveraging MAGICdb predictions of ASCVD status were found to be as efficient as predictions made from traditional lipid blood markers including high-density lipoprotein (HDL) cholesterol, low-density lipoprotein (LDL) cholesterol, and triglycerides. Fecal microbiome sequencing thus represents a paradigm shift in ASCVD prediction, offering a more personalized and function-oriented perspective. It will be exciting to see how these predictions improve when integrated in machine learning algorithms and as more biomarkers get characterized.

MAGICdb performed very well for linking MA metabolism from metagenomic sequencing data to ASCVD disease risk. It would be highly beneficial if databases with similar capabilities could be generated for other keystone gut microbial metabolic processes, such as including bile acid or, in the long term, even complex carbohydrate metabolism. The insights from MAGICdb open the way for new probiotic strategies as well as targeted microbiome interventions aimed at curtailing TMA production levels. In the latter scenario, it would be interesting to investigate the effects of reduced gut bacterial TMA production on the abundance and metabolic capacity of the non-TMA bacteria in the community. Finally, while the pivotal role of TMA in ASCVD is well established, application of MAGICdb presents exciting opportunities to discover novel connections between MA metabolism and other health conditions.
